# Supplementary material for: Enhanced Hybrid Nanogenerator Based on PVDF-HFP and PAN/BTO Coaxially Structured Electrospun Nanofiber
Source: Micromachines (Basel). 2024 Sep 21;15(9):1171. doi: 10.3390/mi15091171 (PMC11433801; doi:10.3390/mi15091171)
Supplement: Supplementary file 1 [file micromachines-15-01171-s001.zip › micromachines-3219662-supplementary.pdf]

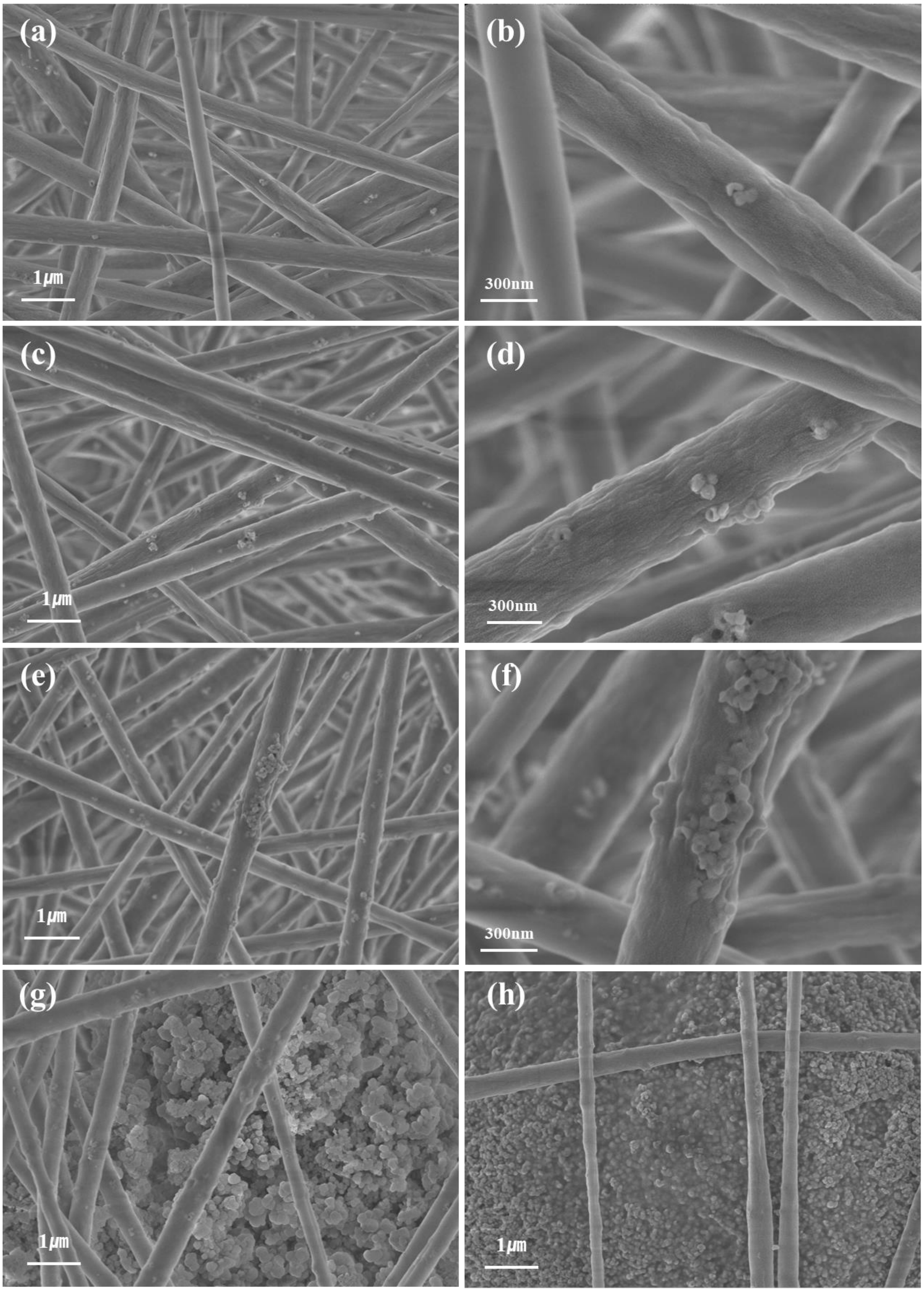

Figure S1. SEM Image of PAN fibers doped with Barium Titanate according to weight percent; (a),(b) 5wt%, (c),(d) 10wt%, (e),(f) 15wr%, (g),(h) 20wt%.

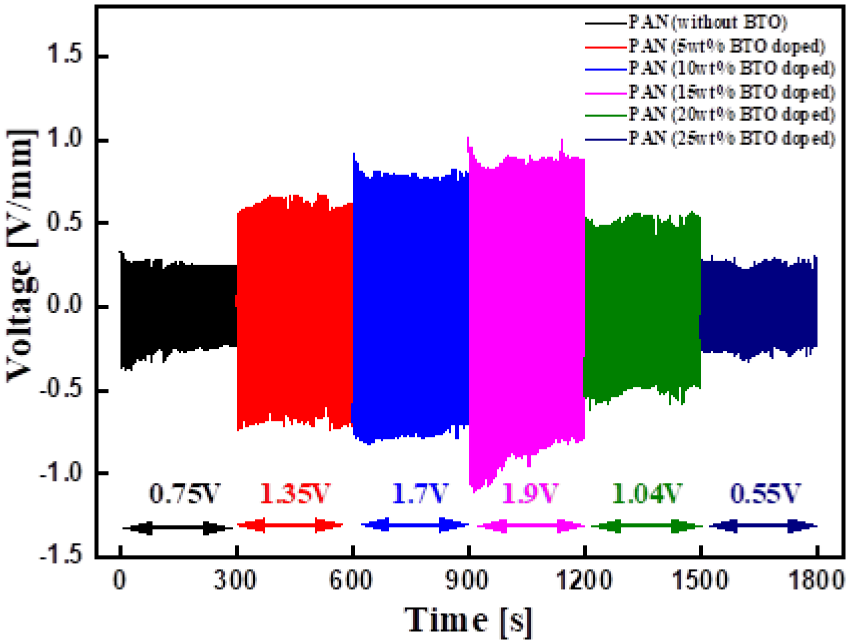

Figure S2. Output voltage of randomly oriented PAN fibers doped with Barium Titanate according to weight percent.

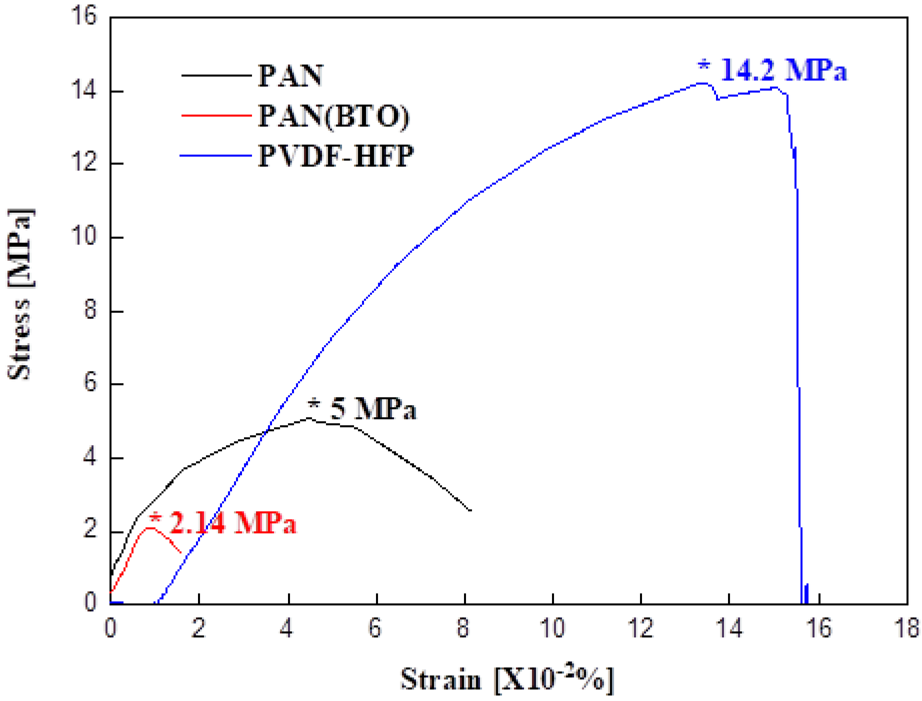

Figure S3. Strain stress curve of PVDF-HFP and PAN fibers with 15wt% Barium Titanate doping and without doping.

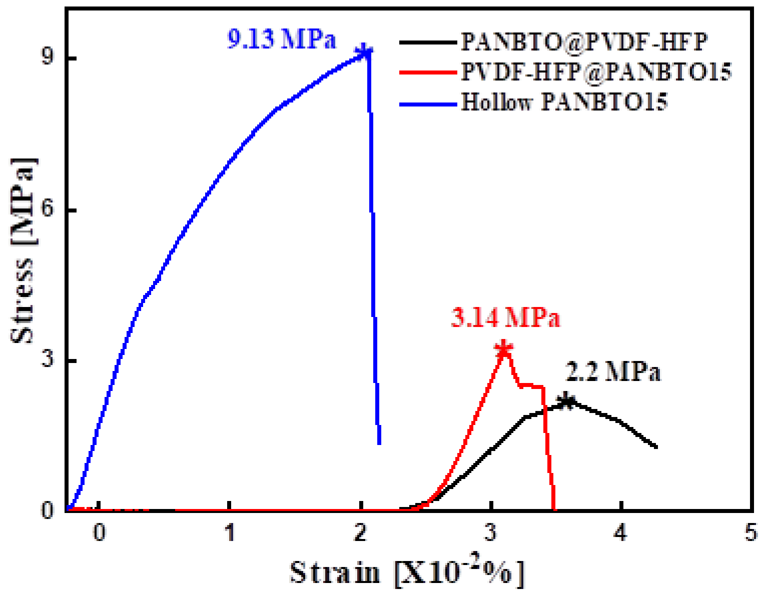

Figure S4. Strain stress curve of coaxially structured fiber; PANBTO@PVDF-HFP, PVDF-HFP@PANBTO, BTO doped PAN hollow fiber.

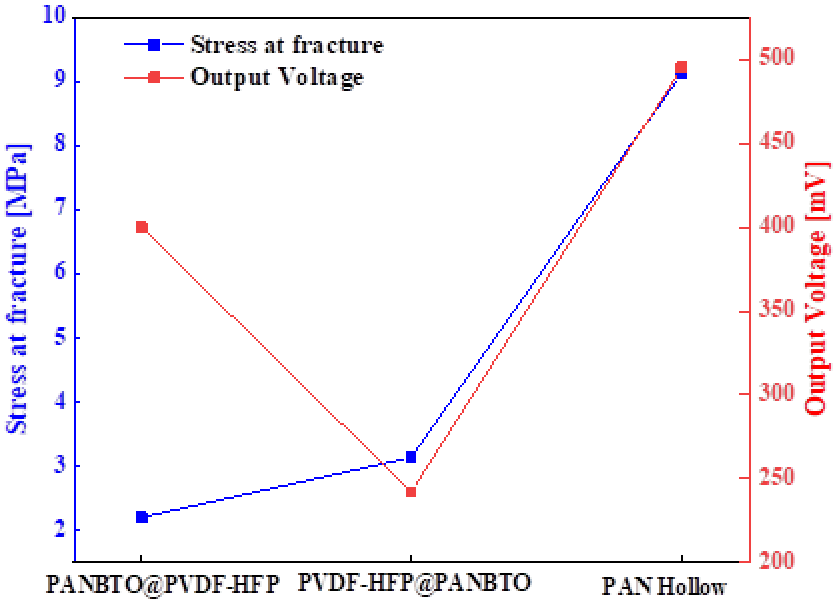

Figure S5. Comparison with mechanical and electrical properties of coaxially structured fiber; PANBTO@PVDF-HFP, PVDF-HFP@PANBTO, BTO doped PAN hollow fiber.
